# Oral Hygiene Practice among Hospitalized Patients: An Assessment by Dental Hygiene Students

**DOI:** 10.3390/healthcare10010115

**Published:** 2022-01-06

**Authors:** Saturnino Marco Lupi, Maurizio Pascadopoli, Carolina Maiorani, Camilla Preda, Benedetto Trapani, Alessandro Chiesa, Francesca Esposito, Andrea Scribante, Andrea Butera

**Affiliations:** 1 Unit of Oral Surgery and Implantology, Section of Dentistry, Department of Clinical, Surgical, Diagnostic and Pediatric Sciences, University of Pavia, 27100 Pavia, Italy; saturninomarco.lupi@unipv.it; 2 Unit of Orthodontics and Pediatric Dentistry, Section of Dentistry, Department of Clinical, Surgical, Diagnostic and Pediatric Sciences, University of Pavia, 27100 Pavia, Italy; andrea.scribante@unipv.it; 3 Unit of Dental Hygiene, Section of Dentistry, Department of Clinical, Surgical, Diagnostic and Pediatric Sciences, University of Pavia, 27100 Pavia, Italy; carolinamaiorani@outlook.it (C.M.); camilla.preda@unipv.it (C.P.); benedetto.trapani01@universitadipavia.it (B.T.); alessandro.chiesa@unipv.it (A.C.); francesca.esposito@unipv.it (F.E.)

**Keywords:** dental hygiene, periodontal disease, systemic diseases, dental education, DMFT, Plaque Index, Marginal Gingival Index

## Abstract

Aim: An epidemiological study was carried out, in hospital wards, with the aim of assessing the oral health status of patients subjected to multiple medical treatments. Material and Methods: The study was conducted at Fondazione IRCCS Policlinico San Matteo (Pavia, Italy). A questionnaire was submitted to patients for the evaluation of oral hygiene devices used; then, a clinical examination was conducted to collect Decayed Missing Filled Teeth (DMFT) index, Plaque Index (PI), and Marginal Gingival Index (MGI) values. Results: Manual toothbrushes were used by a wide range of the sample study (65–100% among hospital wards), together with mouthwash (20–80%); interproximal aids were used by few patients (the lowest recorded value was 33.3%). Conclusion: dental hygienists could be integrated into hospital wards as oral hygiene procedure instructors, for the improvement of the oral health conditions of hospitalized patients.

## 1. Introduction

The well-being of the oral cavity is an integral part of the general state of health of the individual: unfortunately, the increase in world population, and its general aging, has led to an increase in untreated oral diseases [1]. Indeed, data available from the latest Global Burden of Disease shows that unmet demand for dental services reached approximately 3.5 billion cases in 2017; dental caries was the most common condition in the world, with an overall prevalence of 34.1%, while caries in deciduous teeth and severe periodontal disease manifested, respectively, in 7.8% and 7.4% of the global population [2].

Periodontal disease and dental caries are the most widely diffused pathology worldwide [3]: it has been recognized that dental hygiene-therapists could play a role in the diagnosis of these two pathologies, and that their role could be implemented, for example, in public health services, as a support for patients during their hospitalization [4].

The little concern that does exist in the literature for the oral health of hospitalized patients is limited to intensive care unit wards [5]; attention must be paid to the risk of periodontal disease in patients admitted to this ward, as well as to patients in need of mechanical ventilation, where different bacterial colonization develops. Oral care can prevent plaque build-up, and, consequently, reduce the risk of hospital infections related to a low priority of oral hygiene and/or fear of feeling pain; it is important to note that oral care does not bring any benefits, and hospital staff are often poorly trained [6,7]. Some studies, to prevent possible bacterial outbreaks, have found a good efficacy of solutions containing 0.12% chlorhexidine, accompanied by a good brushing of the teeth and washing with iodine solutions [8,9]; in this sense, the nursing staff plays an important role in the maintenance of oral health, and, for this reason, it is beneficial if staffs are effectively instructed by professionals, such as dental hygienists [10]. Furthermore, taking into account the fact that the accumulation of biofilms promotes the development of gingival inflammation, caries, and periodontal disease, and that these worsen during long periods of hospitalization, motivation and education plans should be developed for hospital staff, patients, and caregivers [11]. The oral cavity is a reservoir for pathogens, and accurate oral hygiene, preceded by the screening and removal of potential bacterial foci, can help in managing infections in hospitalized patients [12]. These are patients who can hardly take care of themselves, and therefore trust others for hygienic care, such as oral hygiene maintenance: dental hygienists could be the missing link to help the medical and nursing staff manage the health of these patients [10].

In fact, much medical research is focused on the dental field and oral health, which could be the key to some systemic diseases: clinical evidence has highlighted an association between dental disorders and cardiovascular diseases, as well as diabetes, lung diseases, and obstetric complications [13,14,15,16,17,18]. A certain correlation between diabetes and periodontal disease has been found, as improved clinical conditions and better outcomes of laboratory parameters in diabetic patients have been demonstrated after non-surgical and/or surgical periodontal therapy [14]. A recent study demonstrated that cancer patients show a higher risk of missing teeth and more apical lesions, particularly those of endodontic origin, without root canal fillings [15], although it is advisable to treat dental-periodontal diseases before the onset of treatment, including the extraction of compromised teeth, or, with uncertain prognosis, to motivate and maintain an optimal level of oral hygiene throughout the period of chemo/radiotherapy [16]. During the second and third trimester of pregnancy, there has been noted a frequent onset of periodontal lesions in women; this appears to be associated with some adverse events for pregnancy, such as a reduced increase in the body weight of the unborn child, the development of premature uterine contractions, and the risk of preterm delivery/birth of underweight children [17,18]. Multiple medical treatments, associated with the great variability of commercially available drugs, the continuous evolution of scientific research, the advent of procedures for the treatment of pain, modern drugs for the management of thromboembolic risk, and antibiotic resistance, are elements which it is necessary to respond to with absolute knowledge, training, and basic preparation, to meet the rehabilitation needs in the dental field. For this reason, it is important to provide the clinician, in daily clinical practice, with theoretical and practical support to manage the most common framework of systemic pathology of dental interest, including emergencies, as well as knowledge of prescriptions and the behavior of the most common drugs. The purpose of this work is to present an epidemiological status of the oral hygiene habits of hospitalized patients, and to assess their oral health conditions. In this study, the Decayed Missing Filled Teeth (DMFT) index was recorded for hospitalized patients, together with the Plaque Index (PI) and Modified Gingival Index (MGI), for the assessment of periodontal health.

## 2. Materials and Methods

The survey was conducted by dental hygiene students, under the supervision of tutors, from the the Dental Hygiene Unit, Section of Dentistry, Department of Clinical, Surgical, Diagnostic and Pediatric Sciences, University of Pavia, Italy. One hundred sixty (55% Caucasian, 35% North African, 15% Iberian) patients, from different hospital wards of Fondazione IRCCS Policlinico San Matteo, Pavia, Italy, were interviewed, from October 2018 to April 2019, about their clinical condition and their oral health habits. The study was conducted according to the guidelines of the Declaration of Helsinki and approved by the Unit Internal Review Board (2019-0601).

Patients who agreed to participate to the study were asked to fill in a questionnaire, according to Helsinki regulations. Age and days of hospitalization were recorded, together with the following periodontal and oral health indices, registered in the hospital ward with informed consent: Decayed Missing Filled Teeth (DMFT) [19], Plaque Index (PI) [20], and Modified Gingival Index [21]. The DMFT index was scored by summing up all teeth with restorations, teeth missing at the moment of the survey, and teeth with extensive carious process that would inevitably cause the loss of the tooth (the cause of loss of dental elements, due to caries or periodontal disease, was revealed following the medical history and interview with the patients). The DMFT index was carried out using an oral mirror and dental probe to explore dental surfaces. Each tooth was recorded only once: teeth with caries and restorations, or teeth fractured by caries, were classified as D (decayed); teeth extracted by caries, or teeth candied at extraction, were classified as M (missing); teeth with defective or functional fillings were classified as F (filled). The PI was calculated by assessing the presence or absence of plaque on vestibular, lingual/palatal, and interproximal surfaces. The MGI was calculated using a rating score between 0 and 4, with 0 indicating a tooth with healthy gums, and 4 indicating severe inflammation with spontaneous bleeding. Intra- and inter-operator errors were not assessed, as many professional figures were involved in the present report. In the detection of these indices, it is necessary to take into account possible errors: the effects could be altered according to the general state of health of the patient, some medications taken (such as anticoagulants), or the presence of hard deposits on the surfaces of dental elements.

Medical history was investigated, in particular if patients were suffering from: one systemic disease, more than one disease, or any diseases at all at the moment of the visit. Home oral care habits were investigated, registering the use of manual or electric toothbrushes (or both), interdental aids, and mouthwash.

Collaborating patients who had a caregiver available and any systemic diseases were included in the study, without a reference age range, while non-cooperating patients, patients with psychological problems, patients with oxygen demand, and patients undergoing compulsory health treatment were excluded.

Data analysis was conducted with R software (R version 3.1.3, R Development Core Team, R Foundation for Statistical Computing, Wien, Austria). For each DMFT index, PI, and MGI set of variables, descriptive statistics were calculated. Data included mean, standard deviation, minimum, median, and maximum values for each group. The Kolmogorov–Smirnov test was applied to assess normality of distributions. Subsequently, a repeated measures ANOVA test was performed, followed by Tukey’s test for post-hoc analysis. Significance for all statistical tests was predetermined at *p* < 0.05.

## 3. Results

Average values of periodontal and oral health indices were calculated for patients in each hospital ward (Table 1). The average age of the patients examined was 53–54 years (35% females and 65% males). The highest values were found in the internal medicine ward, where the average hospital stay was around 12 days, a figure doubled in the hematology and surgery departments; increased DMFT index values were observed in the surgery, cardiology, and oncology departments (therefore in patients suffering from gastrointestinal, cardiovascular, and neoplastic problems, such as Crohn’s disease, hypertension, stenosis or heart failure, and cancers in various districts with the possibility of metastasis). Regarding the DMFT index, no statistically significant differences were found among the groups (*p* > 0.05).

The PI was quite high in all hospice wards; this explains the need for constant motivation. A statistically significant difference was found between the PIs of hematology and gynecology (*p* < 0.05).

Regarding MGI, significant differences were found between gynecology and hematology, gynecology and cardiology, and oncology and cardiology (*p* < 0.05).

Percentages of patients with one, more than one, and no diseases are shown in Table 2. In hospital wards, patients have a complex systemic picture: most of them suffer from different pathologies (>1), such as arterial hypertension (data extended to most patients), hyper/hypothyroidism (frequent in gynecology), diabetes, and gastrointestinal problems (mainly in the medical clinic department).

The percentage use of dental aids for oral hygiene is shown in Table 3. Patients mainly used a manual toothbrush (the totality of the patients examined in the medical clinic), while an electric toothbrush was frequently used by patients in the hematology department. Knowledge of the use of dental aids within the hospital population is still limited: the gynecology and cardiology departments displayed the greatest use of toothbrushes and/or dental floss. Finally, some patients used mouthwash at the end of their oral hygiene practices.

## 4. Discussion

From the analysis conducted in the present epidemiological study, patients in the abovementioned hospital wards presented high Gingival Index values, exhibiting gingival inflammation. Surely, the use of suitable aids for the maintenance of oral hygiene could improve this clinical frame. Most of the patients studied used a manual toothbrush, which can be associated with a more difficult brushing of palatal/lingual surfaces; in addition, the frequency of use of interproximal aids should be increased, as residual plaque in the same areas promotes the onset of an inflammatory state, as well as a greater risk of onset of interproximal caries; however, since the relationship between gingival inflammation, or DMFT, and the use of dental aids has not been investigated, it is impossible to conclude that the use of a manual toothbrush causes gingival inflammation or caries. Providing detailed oral health guidance is critical for these patients, where bacterial proliferation must be handled promptly: the attending healthcare professional must be aware of all the risks associated with poor oral health status, and how this may interfere with the systemic health of patients.

The DMFT index recorded between the various departments ranged from 5 to 16.08, and the lowest value was found in the internal medicine ward. The Plaque Index was quite high in all hospital wards (from 52% to 90%), with the exception of gynecology and surgery (52% and 62% respectively). This discovery highlights the need for strong motivation, and is in agreement with some studies in the literature, which emphasized worsening oral conditions during hospitalization, with the increase of oral biofilm [22,23,24,25]. As for patients’ oral hygiene procedures, a large number used a manual toothbrush (from 66% to 100% of patients admitted, especially in oncology). Interdental aids and mouthwash were widely used (from 38% to 61.9% for interdental aids, and from 40.9% to 80% for mouthwash). Unfortunately, no similar studies have emerged in the literature, so there are no data available to compare with the present study, regarding the detection of these indices in patients hospitalized in the same age range, nor are there any studies that clarify and evaluate the activity of the dental hygienist in hospital wards as an aid for oral health. Thus, even on the basis of the limits set out above, the results of this study cannot be implemented in the whole population.

However, knowing that hospitalized patients can have more difficulties in performing correct oral hygiene procedures, the role of a dental hygienist could be that of teaching and monitoring patients during their bed stay. Often, in fact, the maintenance of patients’ oral health is performed by nursing staff, who are poorly trained in the field [26]. The importance of quantifying hospitalized patients’ periodontal and dental status during oral hygiene procedures is highlighted by the findings of the present study; together with systemic health, oral health must be considered to achieve the aim of maintaining a correct balance of oral and systemic microbiomes [27,28].

Although few data are available in the literature, oral health worsens during hospitalization for several reasons. Many patients, in fact, become dependent on hospital staff for care and oral hygiene, which is usually poorly prepared, and has no data available (especially for patients in the intensive care unit), thus favoring the deterioration of oral conditions [22]. Some studies have shown an increase in plaque and gingival inflammation after even short periods of hospitalization, as well as an increase in the Bleeding on Probing index [23,24].

These results reinforce the need for developing oral hygiene protocols and the inclusion of dental professionals in the hospital team, both to provide proper care to patients who are dependent on medical figures, and as a preventative measure for all independent patients, to whom dental professionals could offer instruction on correct oral hygiene methods. In fact, including oral health care programs is effective in the prevention of pneumonia [10], reducing the pain of oral mucositis in patients undergoing chemotherapy [29], allowing early detection of caries in patients with severe hematologic pathologies (which could aggravate the risk of atherosclerosis) [30], facilitating the screening of patients with diabetes for the prevention of inflammation [31], and reducing the risk of developing cardiovascular disease [32]. In addition, hospitalized patients may be directly or indirectly exposed to factors that may impair their oral health status, and the difficulty of maintaining proper oral hygiene during hospitalization worsens this risk [33].

The need for correct oral hygiene in pregnant patients is essential during the first trimester to prevent possible oral complications; the use of an electric toothbrush can reduce the Plaque Index and prevent strong gingivitis. Patients must be treated without stress, kept in a sitting position, and allowed to often change their position. In addition, pregnant patients often experience nausea and vomiting, as well as gastroesophageal reflux; therefore, it is good to motivate patients by providing instructions on both food and the use of fluoride toothpastes, to prevent the risk of caries [34,35,36]. Subjects with coagulation disorders require medical advice before undergoing dental interventions that could cause bleeding. Hemophilic patients should be given clotting factors before, during, and after a dental extraction, or conservative dentistry that requires local anesthesia (for example, fillings); therefore, correct oral hygiene could help in avoiding dental procedures [37,38]. Oncological patients should start dental treatments before the beginning of cancer therapy; professional oral hygiene, the extraction of compromised teeth, and restorations of teeth with wide caries are recommended. At bedtime, it is advisable to use an electric toothbrush and floss (or, in the case of mucosal lesions, a soft toothbrush or sponges soaked in 0.20% chlorhexidine mouthwash). If the patient is subjected to radiation therapy that does not allow them to eat, it is advisable to use a dental rinse and toothpaste with fluoride, to reduce the risk of caries [39,40]. In diabetic patients, evaluation of their periodontal state is needed, and it is influenced by high serum levels of Hba1c. In addition, hyperglycemia causes a thickening of the basal membrane of the capillaries, resulting in a worsening of permeability and perfusion to tissues, oxygenation, and elimination of metabolites from periodontal tissues. These patients may also have xerostomia, acetonemic halitosis, or hyperplastic gingivitis [14,41,42]. Periodontal disease is more aggressive and faster in cardiopathic subjects; moreover, maintaining a balanced oral microbiome is particularly recommended, as it can reduce the complications of cardiovascular diseases. In fact, in the presence of valvular heart diseases and valvular implants, there is a greater risk of endocarditis from the migration of oral bacteria [43,44].

As a matter of fact, dental hygienists should be included in the education of hospitalized and long-term care patients, as they could provide a low-cost approach to prevent the worsening of oral conditions; cooperation with nursing staff and/or caregivers is desirable to completely manage the systemic health of patients [45].

This study, aimed to carrying out an epidemiological analysis of hospitalized patients in order to provide indications for correct maintenance of the oral cavity. However, the results have some limitations, as the data were related to the situation: patients were bedridden, making it impossible to perform a standardized and long-term follow-up, especially in patients with different symptoms; these patients should also be assessed on the basis of the medications taken; finally, patients did not use identical home hygiene devices, which rendered comparing data difficult. Furthermore, it should be taken into account that data were collected by different operators. Samples were not homogeneous, and were derived from different hospital wards. Patients with caregivers were not screened. Another limitation of the study is that data regarding patients’ oral health and conditions of loss of dental elements due to caries or periodontal disease, was collected following the medical history and the interview with the patients; therefore, reporting bias cannot be completely eliminated.

Moreover, multicenter studies are needed to validate the results of the present study. In fact, different protocols of oral hygiene assistance to patients could be adopted by nurses and health workers in charge of patients’ care, depending on different hospital wards and different hospitals; therefore, oral health conditions may change according to the samples considered.

## 5. Conclusions

These preliminary results indicate how difficult it is to maintain good oral hygiene practices in hospitalized patients, highlighting an increase in epidemiological indices, such as the Plaque Index. The control of biofilm would aid in the prevention of oral pathologies, such as carious lesions and gingival pathologies, which are related to some systemic pathologies. Taking this into account, it would be useful to implement oral prevention programs in hospitals, and to instruct health professionals on the proper management of the oral cavity. Including a dental hygienist on the hospital ward staff could help in improving the oral health status of hospitalized patients, as well as intercepting potential dental conditions that deserve treatment as soon as possible. This study shows a high degree of need on the part of hospitalized patients to be followed from the point of view of oral hygiene; however, the results of this study are difficult to generalize due to the intrinsic differences between various hospital structures.

## Figures and Tables

**Table 1:** Descriptive statistics (mean ± standard deviation) of hospitalized patients for each hospital ward.

Hospital Ward	N	Age (y)	Duration of Hospital Stay (d)	DMFT (%)	PI (%)	MGI
Gynecology	21	54.00 ± 13.04	7.67 ± 15.99	10.05 ± 8.80 ^a^	52.00 ± 0.37 ^d^	0.29 ± 0.56 ^f^
Hematology	26	56.46 ± 17.08	14.19 ± 12.88	10.57 ± 10.28 ^a^	80.00 ± 0.28 ^c^	1.08 ± 1.06 ^e,g^
Oncology	34	62.94 ±13.06	10.94 ± 20.27	14.91 ± 9.94 ^a^	76.00 ± 0.24 ^c,d^	0.50 ± 0.66 ^f,g^
Internal Medicine	5	71.60 ± 10.53	6.80 ± 5.36	5.00 ± 5.13 ^a^	90.00 ± 0.22 ^c,d^	0.20 ± 0.00 ^e,f^
Cardiology	44	12.33 ± 6.61	6.72 ± 16.08	11.25 ± 17.09 ^a^	31.84 ± 1.65 ^c,d^	0.67 ± 12.33 ^e^
Surgery	24	16.37 ± 7.79	6.72 ± 14.92	11.44 ± 64.00 ^a^	0.42 ± 0.92 ^c,d^	0.88 ± 16.37 ^e,f^

Legend: for each variable collected, means with the same letters denote no significantly different means (*p* > 0.05). N—number of included patients; y = age of included patients; d = days of hospitalization up to the day of the target examination; DMFT = Decayed Missing Filled Teeth index; PI = Plaque Index; MGI = Modified Gingival Index.

**Table 2:** Presence or absence of one or more diseases for patients in each hospital ward (percentages).

Hospital Ward	No Concomitant Systemic Diseases	Patients Suffering from One Disease	Patients Suffering from More than One Disease
Gynecology	71.5	19	9.5
Hematology	42.3	50	7.7
Oncology	70.6	14.7	14.7
Internal Medicine	0	20	80
Cardiology	100	0	0
Surgery	45.8	41.7	12.5

**Table 3:** Use of dental aids for oral hygiene by patients in each hospital ward (percentages).

Hospital Ward	Manual Toothbrush (%)	Electric Toothbrush	Both Toothbrushes	Interdental Aids	Mouthwashes
Gynecology	66.6	19	14.3	61.9	61.9
Hematology	76.1	26.9	0	42.3	42.3
Oncology	82.4	14.7	2.7	38.2	55.9
Internal Medicine	100	0	0	60	80
Cardiology	75	18.25	7.6	33.3	58.3
Surgery	87.5	12.5	0	33.3	58.3

## Data Availability

The data presented in this study are available on request from the corresponding author.

## References

[1] Peres M.A., Macpherson L.M.D., Weyant R.J., Daly B., Venturelli R., Mathur M.R., Listl S., Celeste R.K., Guarnizo-Herreño C.C., Kearns C. (2019). Oral diseases: A global public health challenge. Lancet.

[2] Bernabe E., Marcenes W., Hernandez C.R., Bailey J., Abreu L.G., Alipour V., Amini S., Arabloo J., Arefi Z., GBD 2017 Oral Disorders Collaborators (2020). Global, Regional, and National Levels and Trends in Burden of Oral Conditions from 1990 to 2017: A Systematic Analysis for the Global Burden of Disease 2017 Study. J. Dent. Res..

[3] Nazir M.A. (2017). Prevalence of periodontal disease, its association with systemic diseases and prevention. Int. J. Health Sci..

[4] Moradi G., Mohamadi Bolbanabad A., Moinafshar A., Adabi H., Sharafi M., Zarei e B. (2019). Evaluation of Oral Health Status Based on the Decayed, Missing and Filled Teeth (DMFT) Index. Iran J. Public Health.

[5] Richards D. (2015). Hygiene-therapists could be used to screen for dental caries and periodontal disease. Evid. Based Dent..

[6] Da Silva J.L., de OEl Kadre G.D., Kudo G.A., Santiago JFJunior Saraiva P.P. (2016). Oral Health of Patients Hospitalized in the Intensive Care Unit. J. Contemp. Dent. Pract..

[7] Brown E.J. (2016). Dental Hygienist Providers in Long-Term Care: Meeting the Need. J. Evid. Based Dent. Pract..

[8] Conley P., McKinsey D., Graff J., Ramsey A.R. (2013). Does an oral care protocol reduce VAP in patients with a tracheostomy?. Nursing.

[9] El-Rabbany M., Zaghlol N., Bhandari M., Azarpazhooh A. (2015). Prophylactic oral health procedures to prevent hospitalacquired and ventilator-associated pneumonia: A systematic review. Int. J. Nurs. Stud..

[10] Coker E., Ploeg J., Kaasalainen S., Carter N. (2017). Observations of oral hygiene care interventions provided by nurses to hospitalized older people. Geriatr. Nurs..

[11] Carrilho Neto A., De Paula Ramos S., Sant’ana A.C., Passanezi E. (2011). Oral health status among hospitalized patients. Int. J. Dent. Hyg..

[12] Lee Y.J., Noh H.J., Han S.Y., Jeon H.S., Chung W.G., Mun S.J. (2019). Oral health care provided by nurses for hospitalized patients in Korea. Int. J. Dent. Hyg..

[13] Dietrich T., Webb I., Stenhouse L., Pattni A., Ready D., Wanyonyi K.L., White S., Gallagher J.E. (2017). Evidence summary: The relationship between oral and cardiovascular disease. Br. Dent. J..

[14] Lalla R.V., D’Ambrosio J.A. (2001). Dental management considerations for the patients with diabetes mellitus. J. Am. Dent. Assoc..

[15] Willershausen I., Schmidtmann I., Azaripour A., Kledtke J., Willershausen B., Hasenburg A. (2019). Association between breast cancer chemotherapy, oral health and chronic dental infections: A pilot study. Odontology.

[16] Brennan M.T., Elting L.S., Spijkevert F.K.L. (2010). Systematic reviews of oral complications from cancer therapies. Oral Care Study Group, MASCC/ISOO: Methodology and quality of the literature. Support Care Cancer.

[17] Muwazi L., Rwenyonyi C.M., Nkamba M., Kutesa A., Kagawa M., Mugyenyi G., Kwizera G., Okullo I. (2014). Periodontal conditions, low birth weight and preterm birth among postpartum mothers in two tertiary health facilities in Uganda. BMC Oral Health.

[18] Komine-Aizawa S., Aizawa S., Hayakawa S. (2019). Periodontal diseases and adverse pregnancy outocomes. J. Obstet. Gynaecol. Res..

[19] Marcinkowski A., Ziebolz D., Kleibrink B.E., Weinreich G., Kamler M., Teschler H., Sommerwerck U. (2018). Deficits in oral health behavior and oral health status in patients after lung transplantation. Clin. Respir. J..

[20] O’Leary T.J., Drake R.B., Naylor J.E. (1972). The plaque control record. J. Periodontol..

[21] Tobias G., Spanier A.B. (2020). Modified Gingival Index (MGI) Classification Using Dental Selfies. Appl. Sci..

[22] Albuquerque B.N., Araújo M.M., Silva T.A., Cota L.O.M., Cortelli S.C., Costa F.O. (2018). Periodontal Condition and Immunological Aspects of Individuals Hospitalized in the Intensive Care Unit. Braz. Dent. J..

[23] Sjögren P. (2011). Hospitalisation associated with a deterioration in oral health. Evid. Based. Dent..

[24] Sachdev M., Ready D., Brealey D., Ryu J., Bercades G., Nagle J., Borja-Boluda S., Agudo E., Petrie A., Suvan J. (2013). Changes in dental plaque following hospitalisation in a critical care unit: An observational study. Crit. Care.

[25] Needleman I., Hyun-Ryu J., Brealey D., Sachdev M., Moskal-Fitzpatrick D., Bercades G., Nagle J., Lewis K., Agudo E., Petrie A. (2012). The impact of hospitalization on dental plaque accumulation: An observational study. J. Clin. Periodontol..

[26] Nagarakanti S., Avuluri J., Chava V.K. (2019). Evaluation of Nurses’ Attitude toward the Provision of Oral Hygiene Care to Hospitalized Patients at Two Private Hospitals in South India. Iran J. Nurs. Midwifery Res..

[27] Butera A., Gallo S., Maiorani C., Molino D., Chiesa A., Preda C., Esposito F., Scribante A. (2020). Probiotic Alternative to Chlorhexidine in Periodontal Therapy: Evaluation of Clinical and Microbiological Parameters. Microorganisms.

[28] Butera A., Lovati E., Rizzotto S., Segù M., Scribante A., Lanteri V., Chiesa A., Granata M., Ruggero R.Y.B. (2021). Professional and Home-Management in non -surgical periodontal therapy to evaluate the percentage of glycated hemoglobin in type 1 diabetes patients. Int. J. Clin. Dent..

[29] Haghighi A., Shafipour V., Bagheri-Nesami M., Gholipour Baradari A., Yazdani Charati J. (2017). The impact of oral care on oral health status and prevention of ventilator-associated pneumonia in critically ill patients. Aust. Crit. Care.

[30] Pauliina U., Jakob P., Joda T., Weiger R., Matti M., Tuomas W. (2020). Oral disorders in patients with newly diagnosed haematological diseases. Clin. Oral Investig..

[31] Simon L.E., Karhade D.S., Tobey M.L. (2020). Oral Health Status of Hospitalized Patients With Type 2 Diabetes. Diabetes Spectr..

[32] Molania T., Malekzadeh Shafaroudi A., Taghavi M., Ehsani H., Moosazadeh M., Haddadi A., Gholizadeh N., Salehi M. (2021). Oral health-related quality of life (OHRQoL) in cardiovascular patients referring to Fatima Zahra Hospital in Sari, Iran. BMC Oral Health.

[33] Terezakis E., Needleman I., Kumar N., Moles D., Agudo E. (2011). The impact of hospitalization on oral health: A systematic review. J. Clin. Periodontol..

[34] Lida H. (2017). Oral health interventions during pregnancy. Dent. Clin. N. Am..

[35] Naaval S., Brickhouse T.H., Shahid H., Smith K. (2019). Factors associated with preventive dental visits before and during pregnancy. J. Womens Health.

[36] Körner P., Georgis L., Wiedemeier D.B., Attin T., Wegehaupt F.J. (2021). Potential of different fluoride gels to prevent erosive tooth wear caused by gastroesophageal reflux. BMC Oral Health.

[37] Shastry S.P., Kaul R., Baroudi K., Umar D. (2014). Hemophilia A: Dental considerations and management. J. Int. Soc. Prev. Community Dent..

[38] Freedman M., Dougall A., White B. (2009). An audit of a protocol for the management of patients with hereditary bleeding disorders undergoing dental treatment. J. Disabil. Oral Health.

[39] Devi S., Singh N. (2014). Dental care during and after radiotherapy in head and neck cancer. Natl. J. Maxillofac. Surg..

[40] Horst J.A., Tanzer J.M., Milgrom P.M. (2018). Fluorides and Other Preventive Strategies for Tooth Decay. Dent. Clin. N. Am..

[41] Frantzis T.G., Reeve C.M., Brown A.L. (1971). The ultrastructure of capillary basement membranes in the attached gingiva of diabetic and nondiabetic patients with periodontal diseases. J. Periodontol..

[42] Sudhakaran S., Surani S.R. (2015). Guidelines for perioperative management of the diabetic patient. Surg. Res. Pract..

[43] Samulak-Zielinska R., Dembowska E., Lizakowski P. (2019). Dental treatment of post-myocardial infarction patients: A review of the literature. Dent. Med. Probl..

[44] Warburton N.J., Caccamese J. (2006). Valvular heart disease and heart failure: Dental management considerations. Dent. Clin. N. Am..

[45] Haumschild M.S., Haumschild R.J. (2009). The importance of oral health in long-term care. J. Am. Med. Dir. Assoc..

